# Multi-responsive upconversion/organic porous silicon nanocomposite for controlled drug release *via* NIR irradiation and tumor microenvironment stimuli

**DOI:** 10.1039/d5ra07668d

**Published:** 2026-01-23

**Authors:** Xiaotao Wang, Zhihao Bi, Yonggui Liao, Chak-Yin Tang, Xuefeng Li, Wing-Cheung Law

**Affiliations:** a Hubei Provincial Key Laboratory of Green Materials for Light Industry, Collaborative Innovation Center for Green Light-weight Materials and Processing, School of Materials Science and Engineering, Hubei University of Technology Wuhan 430068 China; b Key Laboratory for Material Chemistry for Energy Conversion and Storage, Ministry of Education, Hubei Engineering Research Center for Biomaterials and Medical Protective Materials, Hubei Key Laboratory of Material Chemistry and Service Failure, School of Chemistry and Chemical Engineering, Huazhong University of Science and Technology Wuhan 430074 China; c Department of Industrial and Systems Engineering, The Hong Kong Polytechnic University Hung Hom, Kowloon Hong Kong China roy.law@polyu.edu.hk

## Abstract

We report the synthesis of an organic/inorganic architecture, a rare-earth upconversion nanoparticle (UCNP)-based core with organic porous silicon shell nanocapsules, that enables visible and ultraviolet light emission under 980 nm near-infrared (NIR) irradiation for photodegradation. A mesoporous organosilica shell containing disulfide bonds was uniformly coated onto UCNP cores *via* a combined sol–gel and hard-template strategy. Glutathione (GSH) – triggered degradation of the nanocarriers was quantified using the molybdenum blue method. A photoresponsive azobenzene derivative, activatable by both UV and visible light, was synthesized and grafted onto the nanocarrier surface. Doxorubicin (DOX) was loaded into the nanocapsules, achieving a drug-loading capacity of 12.2 wt%. At neutral pH, minimal DOX leakage occurred; maximal release was observed under a composite stimulus environment. The Baker-Lonsdale model was employed to calculate diffusion coefficients under varying release conditions, providing quantitative guidance for designing multi-responsive drug-release systems. *In vitro* cellular assays showed that synergistic stimuli—NIR irradiation combined with an acidic, GSH-rich microenvironment—trigger controllable DOX release, enabling efficient tumor cell ablation. This work demonstrates a versatile, multi-responsive delivery platform combining UCNP-based photoreactivity, GSH-triggered degradation, and photoactive surface gating to achieve targeted, low-toxicity, on-demand chemotherapy.

## Abstract 2

We developed UCNP@organosilica nanocapsules with GSH-triggered degradation and photoresponsive feature for on-demand DOX release. NIR/pH/GSH stimuli enable controlled drug release for targeted, low-toxicity chemotherapy and tumor cell ablation.

## Graphical abstract

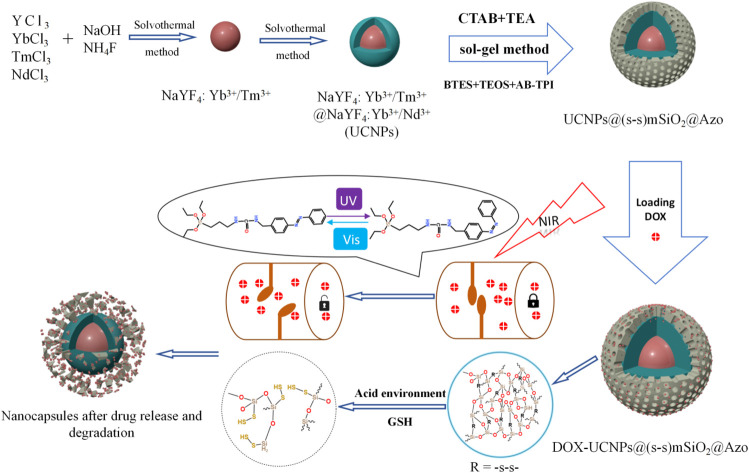

## 1. Introduction

Stimuli-responsive nanodrug delivery platforms undergo reversible or irreversible physical and chemical changes-such as dissolution, precipitation, decomposition, conformational changes, or alterations in chemical structure or assembly morphology under endogenous stimuli (*e.g.*, pH,^1–3^ heat^4^) or exogenous stimuli (*e.g.*, light,^5–7^ ultrasound^8,9^). Due to their unique characteristics, these platforms hold significant potential for tumor therapy and diagnosis.^2,10–12^ Thus, designing a stimuli-responsive platform that enables precise drug release at targeted sites in response to multiple factors can enhance biocompatibility while improving drug release efficiency. However, most photoresponsive units (*e.g.*, *o*-nitrobenzyl derivatives,^13–15^ coumarin derivatives,^16,17^ thymine,^18^ azobenzene derivatives,^19–21^ spiropyran^22,23^) rely on ultraviolet (UV) light, whose toxicity and low tissue penetration limit biomedical applications.

In recent years, near-infrared (NIR) responsive materials have been extensively studied due to their deeper tissue penetration and lower toxicity.^24–28^ Upconversion nanoparticles (UCNPs), with their unique optical properties, could absorb NIR light to emit UV and visible light. The UV light could make most photosensitive groups to form the basis for controlled drug release through the variation of size or dipole moment,^28–30^ while the visible light could synergize with imaging equipment for potential bioimaging applications.^31,32^ UCNPs could serve as an excellent converter for NIR light-activated drug delivery platforms.

Mesoporous silica nanoparticles (MSNs), as representative silicon-based materials, demonstrate immense potential for disease treatment owing to their synthesis simplicity, structural controllability, large specific surface area, and ease of functionalization and modification.^33–35^ Combining MSNs with UCNPs enhances the biocompatibility and drug-loading capacity of UCNPs. However, the slow biodegradation of pure inorganic Si–O–Si structures (degradation typically requiring months) severely hinders the clinical translation of MSNs.^36–38^ Current research has thus shifted toward fabricating various organic/inorganic hybrid mesoporous organosilica nanoparticles (MONs) by introducing metal coordination bonds, disulfide bonds, and other linkages into Si–O–Si frameworks to improve biodegradability.^39,40^ Nevertheless, drug-loaded MONs often suffered from premature drug leakage during systemic circulation. To address this, multiple strategies have been reported, among which capping with photoresponsive groups is an effective approach.^41^ Azobenzene, a typical photoresponsive unit, exhibits rapid photoinduced *cis*–*trans* isomerization under UV/visible light irradiation. This conformational switching between rod-like and bent states makes azobenzene derivatives ideal candidates for photoresponsive molecular gates.^42^

Herein, we report a nanocomposite particle, UCNPs@(s-s)mSiO_2_, in which the photoresponsive ligand 4-(3-triethoxysilylpropylureido)azobenzene (AB-TPI) is grafted onto the organosilica channels of the nanocapsules to form a light-controlled molecular switch. Upon NIR excitation, the UCNPs emit UV/visible light, triggering reversible *cis*–*trans* isomerization of azobenzene. This process induces periodic changes in pore size, enabling precise light-controlled drug release. Disulfide bonds are introduced on the mesoporous silica surface as redox-responsive units, leveraging the high glutathione (GSH) concentration in the tumor microenvironment to trigger disulfide cleavage enhancing drug release efficiency with low toxicity. This innovative approach aims to enhance the precision and efficacy of drug delivery systems, paving the way for more effective and safer therapeutic interventions.

## 2. Materials and methods

### 2.1 Materials

Lanthanide rare earth oxides were purchased from Shanghai Yuelong rare earth new material Co., Ltd. Tetraethyl orthosilicate (TEOS), cetyltrimethylammonium bromide (CTAB; Sigma-Aldrich, 99%), triethanolamine (TEA), bis[γ-(triethoxysilicon)propyl]-tetrasulfide(BTES). Copper(ii) sulfate, l-ascorbic acid, potassium carbonate (K_2_CO_3_), tetrahydrofuran(THF; Aladdin, 99%) were dried before use using the molecular sieve 4A. Other chemicals including 4-aminoazobenzene (TCI, 98%), 3-(triethoxysilyl)propyl isocyanate (TPI; Sigma-Aldrich, 95%). The 4T1 cells utilized for *in vitro* cell viability assessment were obtained from Shanghai Aolu Biotechnology Co., Ltd and incubated in a laboratory setting. The Taoniu protein serum was purchased from Zhejiang Tianhang Biotechnology Co., Ltd.

### 2.2 Characterizations

The photoluminescence spectrum from 200 nm to 700 nm was measured using a near-infrared excitation spectrometer. The average size and size distribution of nanoparticles were determined by dynamic light scattering (DLS) analysis (90PLUS PALS, Brookhaven, USA), with each sample undergoing at least three consecutive measurements to ensure consistency. Transmission electron microscopy (TEM) images were acquired using a Tecnai G2 microscope (NBT-980-5, Nbet, Beijing) operated at 24.0 kV accelerating voltage. UV-vis absorption characteristics of nanoparticles and adsorbents were investigated with a Hitachi High-Technologies Corporation spectrophotometer (Tokyo, Japan) across a scanning wavelength range of 200–600 nm. Fourier-transform infrared (FTIR) spectra were recorded on a Tensor II spectrometer (Bruker, Germany) with spectral acquisition from 400 to 4000 cm^−1^.

### 2.3 Synthesis of β-NaYF_4_:Yb^3+^/Tm_3_+UCNPs

React rare earth oxides with concentrated hydrochloric acid to form rare earth chlorides. UCNPs were synthesized using the solvothermal method. In a typical procedure, 2.5 mL of YCl_3_ (0.2 M), 2.475 mL of YbCl_3_ (0.2 M), and 50 µL of TmCl_3_ (0.1 M) aqueous solutions were combined in a four-necked flask. The mixture was heated to 70–80 °C under vigorous stirring to remove residual water. Oleic acid (OA) and 1-octadecene (ODE) were then introduced, followed by degassing at 110 °C under nitrogen flow to eliminate moisture. Subsequently, the temperature was elevated to 160 °C and maintained for 1 h to form rare-earth-oleate complexes, yielding a bright yellow transparent solution. After cooling to room temperature, 5 mL of sodium hydroxide (NaOH, 0.5 M in methanol) and 8 mL of ammonium fluoride (NH_4_F, 0.5 M in methanol) were sequentially added under continuous stirring. The mixture was heated to 45 °C and aged for 30 min to crystallize cubic-phase α-NaYF_4_:Yb ^3+^/Tm^3+^. A secondary degassing step was performed at 65 °C for 30 min, after which the reaction system was rapidly heated to 302 °C and held for 90 min to induce phase transformation into hexagonal β-NaYF_4_:Yb^3+^/Tm^3+^ nanoparticles. The resulting nanoparticles were purified by triple centrifugation with ethanol and redispersed in 10 mL of chloroform for subsequent use.

### 2.4 Synthesis of core–shell UCNPs

UCNPs are coated with a NaYF_4_:Yb^3+^/Nd^3+^ shell on NaYF_4_:Yb^3+^/Tm^3+^ in the same manner. Firstly, 2.75 mL (0.2 M) of YCl_3_ aqueous solution, 0.25 mL YbCl_3_ aqueous solution, and 2 mL NdCl_3_ aqueous solution are added to a reactor and heated to remove moisture. Subsequently, oleic acid (OA) and 1-octadecene (ODE) are uniformly mixed and introduced into a four-neck flask. Further dehydration is carried out under vacuum at 110 °C to remove residual moisture. The system is then reacted at 160 °C for 40 minutes to form a complex between rare earth chloride salts and oleic acid molecules, resulting in a bright yellow solution. Next, 9 mL of NaYF_4_:Yb^3+^/Tm^3+^ nanoparticles are added to the reactor and mixed thoroughly at room temperature before heating to remove the solvent. The system is then cooled to room temperature, and 5 mL of sodium hydroxide methanol solution (0.5 M) and 8 mL of ammonium fluoride methanol solution (0.5 M) are added, stirred evenly, and reacted at 45 °C for 30 minutes to form α-NaYF_4_. After the system is heated to 65 °C to remove methanol under vacuum, it is held at 302 °C under a nitrogen atmosphere for 90 minutes, during which the NaYF4:Yb^3+^/Nd^3+^ shell is coated onto the surface of the NaYF_4_:Yb^3+^/Tm^3+^ nanoparticles. The UCNPs are collected by centrifugation, washed three times with anhydrous ethanol, and then dispersed in 10 mL of cyclohexane for later use.

### 2.5 Synthesis of photoresponsive molecules 4-(3-triethoxysilylpropylureido)azobenzene (AB-TPI)

**Scheme 1:**
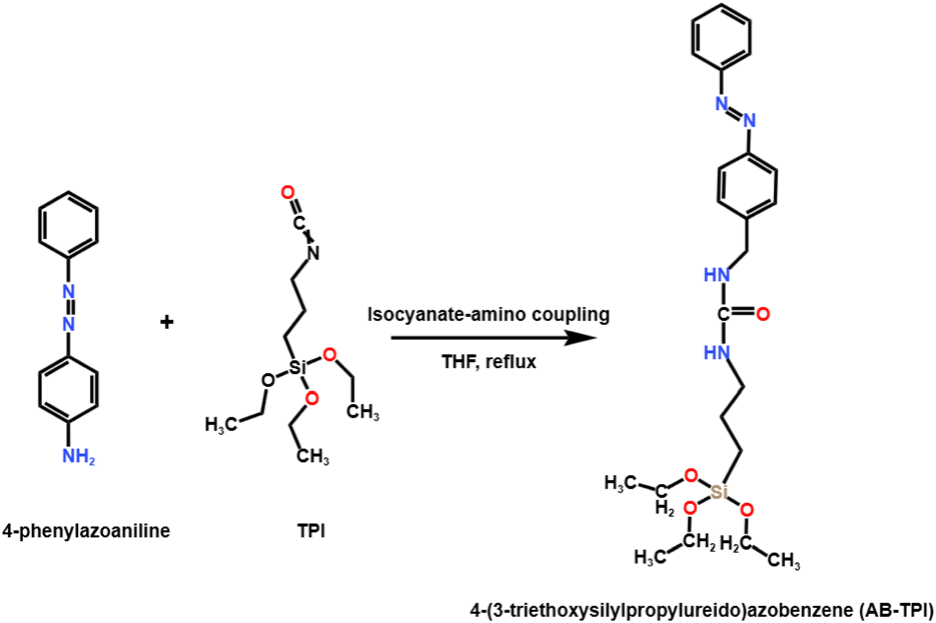
Synthesis path of AB-TPI photosensitive linker.

The photoresponsive molecules molecule 4-(3-triethoxysilylpropylureido)azobenzene (AB-TPI) was prepared by use of the reported method.^43,44^ The experimental process is as shown in Scheme 1: TPI (2.05 g, 8.12 mmol) and 4-aminoazobenzene (1.58 g, 7.85 mmol) were charged into anhydrous THF (12 mL) under a nitrogen atmosphere. The resulting solution was heated under reflux overnight. Subsequently, *n*-hexane (40 mL) was added to the cooled mixture, which was then stored at −20 °C for 12 hours to facilitate crystallization. The solid product underwent three successive washings with *n*-hexane, yielding orange crystals as the final purified material.

### 2.6 Synthesis of UCNP@(s-s)mSiO_2_@Azo

Using synthesized UCNPs as templates, TEOS as the inorganic silicon source, BTES as the organic silicon source, and hexadecyltrimethylammonium bromide (CTAB) as a surfactant, an organic silicon shell layer was coated using the hard-template method. Leveraging chemical homogeneity, a sol–gel process was employed to apply a silica coating. A photosensitive adsorption molecule, AB-TPI, was incorporated during the synthesis. The overall synthesis process is as follows: Initially, 0.2 g of CTAB was dissolved in 25 mL of an aqueous solution and placed in a three-necked flask. Then, 1 mL of the previously prepared UCNPs was added and the mixture was stirred evenly. The solution was sonicated until it became clear and transparent. Subsequently, 1 mL of ethanol was added to the solution, followed by the dropwise addition of 0.1 g of triethanolamine (TEA) and stirred thoroughly. The flask was then placed in an oil bath for the reaction, with the temperature set to 75 °C. A mixture of 2 mL of ethanol containing 90 µL of TEOS, 40 µL of BTES, and 0.004 g of AB-TPI was prepared and added dropwise to the aforementioned solution, followed by continuous stirring for 12 hours. The reaction was then terminated by cooling the solution to room temperature, and the product was collected by centrifugation and washed with 10 mL of anhydrous ethanol. This centrifugation step was repeated three times at a speed of 13 000 rpm for 10 minutes each. The product was then dispersed in 30 mL of ethanol in a three-necked flask and reacted in an oil bath at 75 °C for 4 hours. Then, 0.24 g of ammonium nitrate was added to the system, and the reaction was carried out at 60 °C for 12 hours to remove CTAB from the pores. Finally, the product was centrifuged, and the sample was dried for further use.

### 2.7 Drug loading

Dispersion of 15 mg UCNP@(s-s)mSiO_2_@Azo nanoparticles in 15 mL of doxorubicin (DOX) solution (0.5 mg mL^−1^) was carried out with stirring at room temperature for 24 hours. The reaction solution was collected and centrifuged at 13 000 rpm for 55 minutes. The nanoparticles were washed three times with deionized water to remove excess DOX molecules from the surface of the UCNP@(s-s)mSiO_2_@Azo nanoparticles. The supernatant was collected, and the absorbance at 480 nm was measured using a UV-vis spectrophotometer to calculate the drug loading capacity.

### 2.8 Quantitative detection of organic mesoporous silica degradation by molybdenum silica blue method

Precisely weighed sodium metasilicate pentahydrate was dissolved and volumetrically standardized to prepare a stock solution, from which silicic acid solutions of various concentrations were prepared. Aliquots of each concentration were sequentially treated with ethanol, hydrochloric acid, and oxalic acid to eliminate interference. Subsequently, ammonium molybdate was added to form a yellow silicomolybdate complex, followed by storage in the dark for 20 minutes. Ascorbic acid was then introduced to reduce the complex into a molybdenum blue chromogenic agent. The solution was diluted, and absorbance measurements were performed at 810 nm using a UV-vis spectrophotometer. A silicic acid standard curve was established by plotting the relationship curve between concentration and absorbance. Throughout the experiment, the order of reagent addition and reaction conditions were strictly controlled, and plasticware was rigorously used to prevent the introduction of extraneous silicon sources.

### 2.9 Degradation protocol for UCNP@(s-s)mSiO_2_

To simulate the tumor microenvironment, a phosphate-buffered saline (PBS) solution (pH 5.0) containing 10 mM glutathione (GSH) was used. UCNP@(s-s)mSiO_2_ nanoparticles (3 mg) were encapsulated in a dialysis bag (molecular weight cutoff: 12 kDa), and the dialysis bag was placed into 100 mL of tumor microenvironment-simulated fluid. To avoid introducing additional silicon sources, all containers were made of polypropylene (PP) material. Continuous stirring (200 rpm) was performed on an orbital shaker at 37 °C. The beaker was sealed to minimize evaporation during degradation. Parallel control experiments were conducted under identical conditions. At predetermined time intervals, solutions of the released medium outside the dialysis bag were collected, and the silicic acid content was quantitatively analyzed *via* ultraviolet-visible (UV-vis) spectrophotometry.

The silicon content was determined using the molybdenum blue method. The specific procedure was as follows: 5 mL of the external solution was transferred to a plastic bottle, followed by sequential addition of 8 mL ethanol (as a color development stabilizer), 8 mL 0.1 M HCl (to establish an acidic environment), and 10 mL 5% (w v^−1^) oxalic acid solution (to eliminate phosphate impurities). After shaking thoroughly, 8 mL 5% (w v^−1^) ammonium molybdate solution was added to form a yellow silicomolybdate complex. Subsequently, 5 mL 2% (w v^−1^) ascorbic acid solution was added to reduce the complex to stable silicomolybdenum blue. The absorbance of the silicomolybdenum blue was measured using a UV-vis spectrophotometer for quantitative analysis. Degraded particles in the dialysis bag were collected *via* centrifugation, and morphological and structural changes during degradation were analyzed by transmission electron microscopy (TEM).

### 2.10 Exploration of drug controlled release behavior

UCNP@(s-s)mSiO_2_ (6 mg) were dispersed in 1 mL of pH 7.4 buffer solution, and the mixture was then transferred into a dialysis bag with a molecular weight cutoff of 12 000 kDa. The dialysis bags were placed in 4 mL of buffer solution environments at pH 7.4, pH 5.0, pH 7.4 with 10 mM GSH, and pH 5 with 10 mM GSH under 980 nm laser irradiation, respectively. At regular intervals, 3 mL of the solution outside the dialysis bag was taken out, and the absorption peak intensity of DOX at 480 nm was measured to calculate the released DOX concentration.

### 2.11 *In vitro* cell viability assay

The cell viability of UCNP@(s-s)mSiO_2_@Azo and DOX-UCNP@(s-s)mSiO_2_@Azo nanocapsules was assessed using the MTT assay. The experimental cells selected were the 4T1 mouse breast cancer cell line, which was seeded in a 96-well plate at a density of 1 × 10^5^ cells per mL. The cells were incubated for 24 hours in a humidified atmosphere containing 5% CO_2_ at 37 °C to ensure proper attachment to the wells.

Different concentrations of DOX-UCNP@(s-s)mSiO_2_@Azo drug-loaded nanocapsules were added to each well of the 96-well plate, followed by an additional incubation period of 12 hours. The concentrations of DOX used were as follows: 0.72, 1.46, 2.92, 5.84, and 11.68 µg mL^−1^ (based on equivalent drug loading). The concentrations of UCNP@(s-s)mSiO_2_@Azo nanocomposite particles tested were: 6.25, 12.5, 25, 50, and100 µg mL^−1^. After incubating in a dark environment for another period of twelve hours, the cells were exposed to a laser with a power output of 2 W cm^−2^ at a wavelength of 980 nm for 30 minutes; they then underwent an additional incubation in darkness for 24 hours. The MTT solution (20 µL) was added to each well and further incubated for four hours. Afterward, 150 µL dimethyl sulfoxide (DMSO) was introduced into each well and placed on a low-speed shaker for ten minutes. Finally, the absorbance at 490 nm was measured using an enzyme-linked immunosorbent assay (ELISA) reader.

### 2.12 *In vitro* imaging of DOX-UCNP@(s-s)mSiO_2_@Azo nanocapsules

The process of endocytosis and release of DOX by the complex particles in cells was observed by laser confocal scanning microscopy. To study the cell uptake process, 4 T1 cells were inoculated in 12-well plates (with a cell density of 1 × 10^5^ cells per mL) and incubated in a humid atmosphere of 5% CO_2_ (37 °C) for 24 hours. The cells were incubated with DOX-UCNP@(s-s)mSiO_2_@Azo medium dispersed at the same concentration for 1, 3 and 6 hours, respectively. After the incubation was completed, the cells were thoroughly washed three times using PBS buffer, and then Hoechst 33 342 staining solution was added for 10 minutes of nuclear staining. After rinsing again with PBS, the cell samples were fixed with 4% paraformaldehyde solution, and finally the image acquisition was completed by laser confocal scanning microscopy.

## 3. Results and discussion

### 3.1 Preparation of UCNP@(s-s)mSiO_2_@Azo composite nanoparticles

**Scheme 2:**
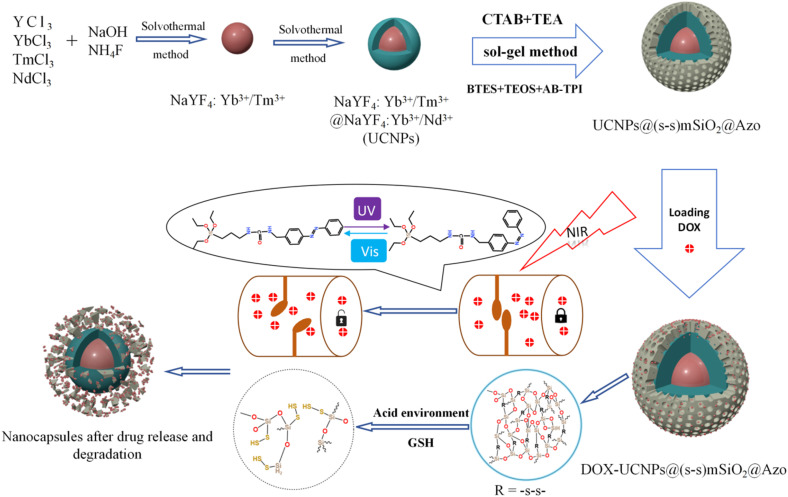
Synthetic route of UCNP@(s-s)mSiO_2_@Azo nanocapsules and loading and release of DOX on the UCNP@(s-s)mSiO_2_@Azo nanocapsules.

**Fig. 1:**
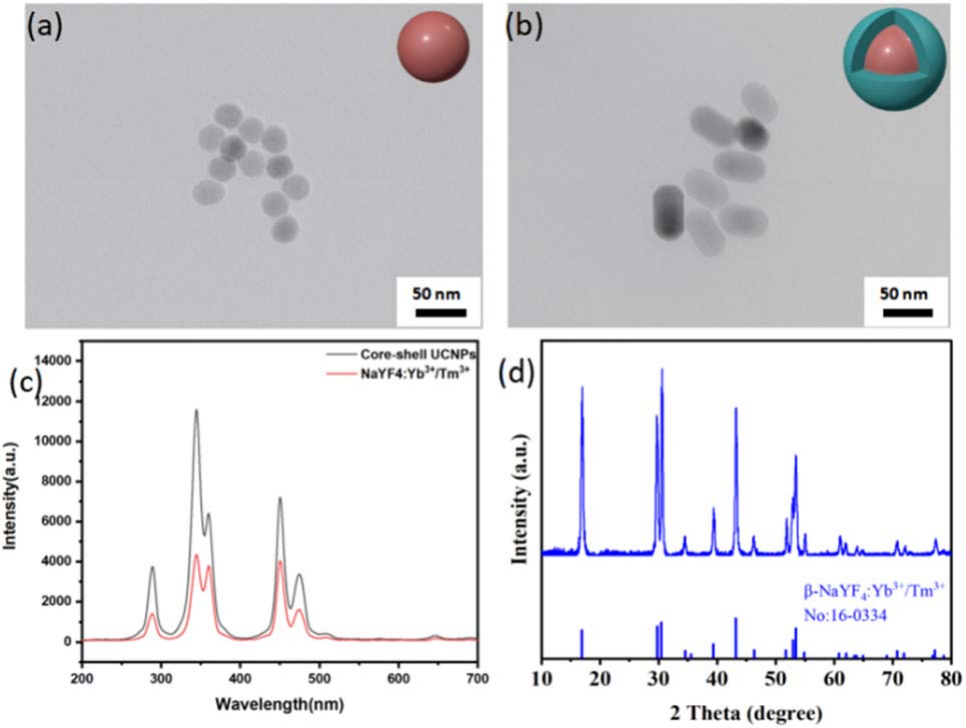
(a) TEM images of NaYF_4_: Yb^3+^/Tm^3+^; (b) TEM images of NaYF_4_: Yb^3+^/Tm^3+^@NaYF_4_:Nb^3+^/Ym^3+^; (c) upconversion luminescence spectrum of NaYF_4_: Yb^3+^/Tm^3+^and NaYF_4_: Yb^3+^/Tm^3+^@NaYF_4:_Nb^3+^/Ym^3+^; (d) XRD pattern of NaYF4: Yb^3+^/Tm^3+^ NPs.

Scheme 2 illustrates the synthetic route of the UCNP@(s-s)mSiO_2_@Azo composite,the loading and controlled release of DOX. To minimize surface quenching effects, enhance luminescence efficiency, an active shell was coated on the core of UCNPs. Core–shell structure has reduced energy loss, and improved surface lattice defects of the particles. Fig. 1a shows that the monodisperse NaYF_4_:Yb^3+^/Tm^3+^ (core) has an average diameter of 26 nm(±2 nm). Fig. 1b depicts core–shell-structured upconversion nanoparticles. The morphology transitions to a rod-like with average dimensions increasing to approximately 43 nm (length) × 26 nm (diameter). The synthesized UCNPs were passivated with oleic acid (OA), to prevent nanoparticle aggregation. Fig. S1a and b displayed the SEM images of NaYF_4_: Yb^3+^/Tm^3+^ and NaYF_4_: Yb^3+^/Tm^3+^@NaYF_4_: Nb^3+^/Ym^3+^ (UCNPs), respectively. The XRD pattern of NaYF_4_: Yb^3+^/Tm^3+^@NaYF_4_:Nb^3+^/Ym^3+^ (Fig. 1d) reveals diffraction peaks are consistent with the hexagonal NaYF_4_ standard, confirming UCNPs with high crystallinity.

The synthesized UCNP cores (NaYF_4_: Yb^3+^/Tm^3+^) and core–shell structures (NaYF_4_: Yb^3+^/Tm^3+^@NaYF_4_: Nb^3+^/Ym^3+^) were dispersed, and their luminescent properties were analyzed by fluorescence spectroscopy under 980 nm laser excitation (1.5 W cm^−2^). Fig. 1c compares the luminescence spectra of the core and core–shell UCNPs at identical concentrations. Under 980 nm excitation, the UCNPs emitted intense ultraviolet peaks at 290 nm, 345 nm, and 360 nm, along with visible blue-ultraviolet peaks at 450 nm and 475 nm. In this work, coating the UCNPs with a shell layer effectively isolates the core from the external environment, reduces surface quenching, and significantly enhances luminescence intensity.

To achieve surface functionalization of UCNPs and facilitate subsequent grafting of photosensitive groups, a surface modification strategy based on hydrochloric acid-assisted ligand removal was employed. As shown in Fig. S2, this approach successfully transformed the hydrophobic UCNPs, initially dispersed in cyclohexane, into a hydrophilic system capable of aqueous dispersion. To further enhance the drug-loading capacity and functionality of UCNPs, a biodegradable organic mesoporous silica shell (MONs) was coated onto the surface of the nanoparticles.

The coating of mesoporous organic silica nanoparticles (MONs) improved the responsiveness of biomolecule release and degradation. Under conditions where the total amount of silica source was kept constant, adjusting the proportion of siloxane precursors significantly influenced dispersion quality. As demonstrated in Fig. S3, reducing the proportion of the organic silica source bis[γ-(triethoxysilyl)propyl]-tetrasulfide (BTES) while maintaining a constant total silica source improved the clarity and dispersion of UCNP@(s-s)mSiO_2_ nanoparticles. Conversely, increasing the proportion of organic silica precursors led to morphological defects in the particles. Optimal particle morphology and dispersion were achieved when the ratio of organic silica source (BTES) to inorganic silica source (tetraethyl orthosilicate, TEOS) was 4 : 9.

**Fig. 2:**
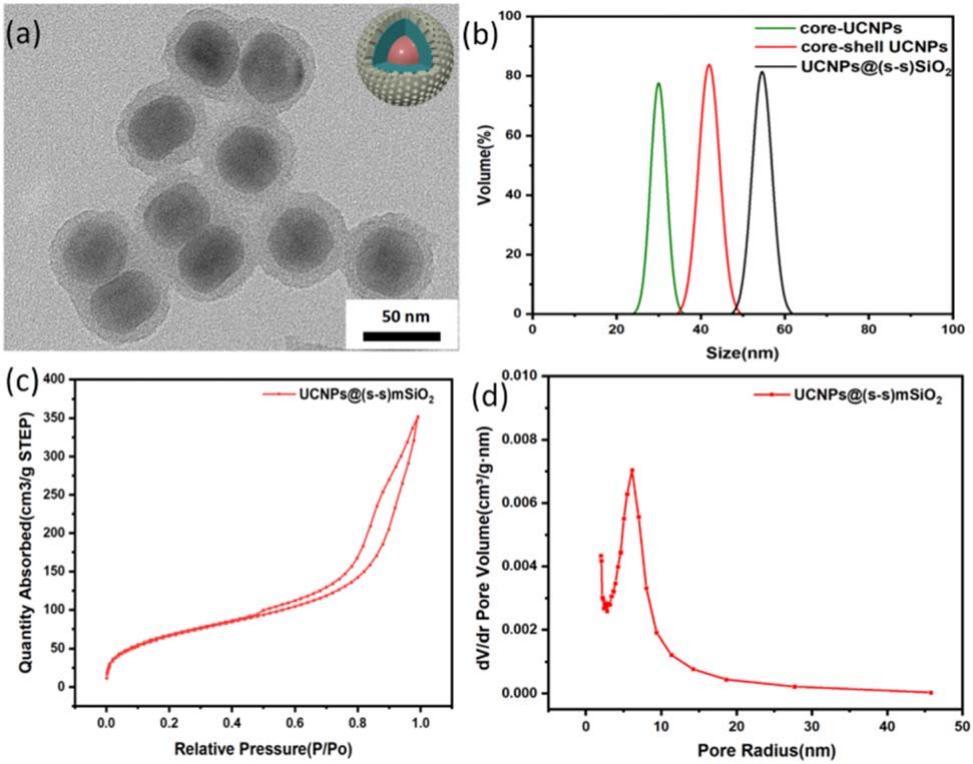
(a) TEM images of UCNPs@(s-s)mSiO_2_; (b) dynamic light scattering particle size distribution, NaYF_4_:Yb^3+^/Tm^3+^, NaYF_4_: Yb^3+^/Tm^3+^@NaYF_4_: Nb^3+^/Ym^3+^(UCNPs) and UCNPs@(s-s)mSiO_2_; (c) nitrogen adsorption–desorption diagram of UCNP@(s-s)mSiO_2_ particles; (d) pore size distribution of UCNP@ (s-s) mSiO_2_ particles.

The particle sizes of UCNPs and UCNP@(s-s)mSiO_2_ nanoparticles were characterized using transmission electron microscopy (TEM) and dynamic light scattering (DLS). TEM images of UCNP@(s-s)mSiO_2_ (Fig. 2a) revealed a thin, uniform mesoporous silica layer coated onto hydrophobic UCNPs *via* a sol–gel method, with a shell thickness of approximately 10 nm. Dynamic light scattering measurements (Fig. 2b) indicated uniform particle sizes of approximately 25 nm for NaYF_4_: Yb^3+^/Tm^3+^, 45 nm for NaYF_4_: Yb^3+^/Tm^3+^@NaYF_4_:Nb^3+^/Ym^3+^(UCNPs), and 56 nm for UCNPs@(s-s)mSiO_2_, consistent with dimensions observed in TEM and SEM images. Fig. 2c and d shows the mesoporous nanoparticles exhibited a specific surface area of 243.99 m^2^ g^−1^, with pore size distribution centered at 44.5 Å (4.45 nm). Hysteresis between adsorption and desorption isotherms in the relative pressure range of 0.5–0.95 confirmed the mesoporous structure of the nanoparticles, which is advantageous for subsequent drug adsorption, loading, and controlled release behavior of DOX.

### 3.2 Photoresponsiveness of UCNPs@(s-s)mSiO_2_@Azo

The synthesized UCNPs@(s-s)mSiO_2_ nanoparticles could be used for drug loading and release but struggle to adapt to complex physiological microenvironments during administration.^37^ Typically administered *via* subcutaneous injection, nanoparticles are prone to rapid clearance and premature drug leakage during systemic circulation. To enhance the drug-loading capacity of UCNPs and improve the controlled release efficiency of DOX, a photoresponsive adsorbent AB-TPI was synthesized by an amino coupling reaction with 3-(triethoxysilyl)propyl isocyanat. The reactant and products were characterized by UV visible spectrophotometer (Fig. S4a) and FTIR (Fig. S4b).

**Fig. 3:**
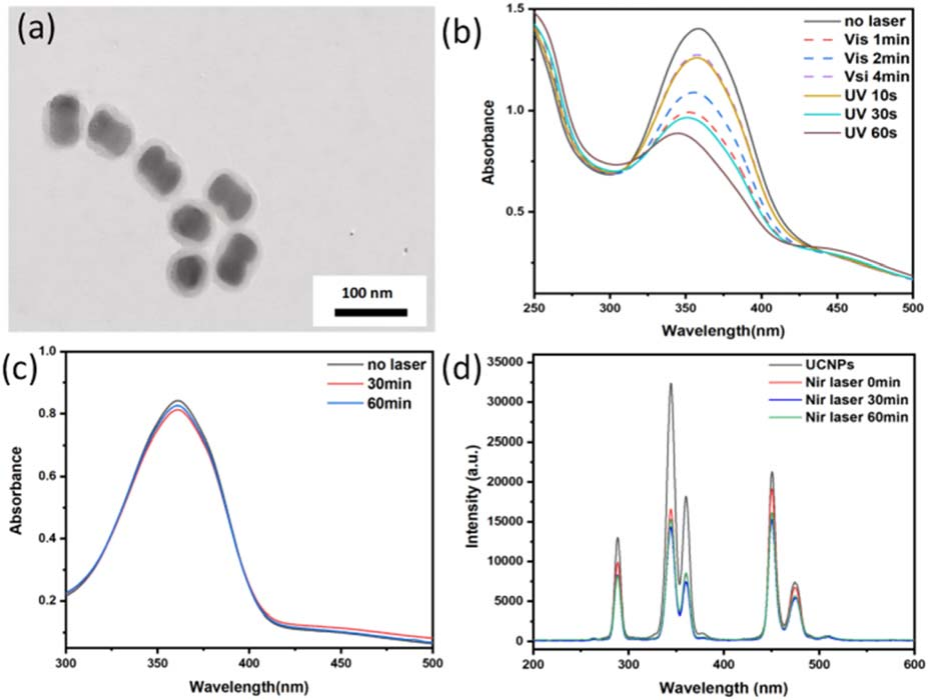
(a)TEM images of UCNPs@(s-s)mSiO_2_@Azo; (b) UCNPs@(s-s)mSiO_2_@Azo photoisomeric UV-vis luminescence spectra; (c) UCNPs@(s-s)mSiO_2_@Azo UV-vis absorption spectra before and after 980 nm NIR irradiation for 0.5 h–1 h; (d) UCNPs@(s-s)mSiO_2_@Azo the emission spectra were measured before and after 980 nm NIR irradiation for 0.5 h–1 h.

UV and visible light efficiently promote *trans* to *cis* photoisomerization of azobenzene. AB-TPI was successfully grafted into the mesopores of UCNPs@(s-s)mSiO_2_*via* a sol–gel method, functioning as both a drug gatekeeper and molecular stirrer.^24^ Comparison of Fig. 2a and 3a indicates no significant morphological changes to the nanoparticle surfaces after AB-TPI grafting. To verify whether the photo-responsive groups grafted on the surface, the UCNPs@(s-s)mSiO_2_@Azo sample was irradiated with UV and visible light, and its UV-visible absorption spectrum was recorded (Fig. 3b). The spectral results confirm that the azobenzene moieties could reversibly isomerize between the *trans* and *cis* configurations. From Fig. 3d, during NIR irradiation (0–1 h), UV and blue light emissions from UCNPs are effectively absorbed by azobenzene. The absorbed UV light supported the isomerization from *trans*–*cis* structure while visible light make *cis* restored to *trans* structure. After 60 min the isomerization from *trans* to *cis* and the *cis* to *trans* reached dynamic balance. Similar NIR induced dynamic balance transformation was also proved.^45,46^ The continuous *cis*–*trans* isomerization of azobenzene within the pores create molecular stirrer effect, providing a theoretical foundation for light-controlled drug release in photoresponsive drug-loaded composite particles.

### 3.3 Kinetic analysis of glutathione degradation

The tumor microenvironment is characterized by high glutathione (GSH) levels and a weakly acidic pH. Disulfide bonds are well-documented to undergo redox reactions with high-concentration GSH,^39,47^ leading to bond cleavage. By incorporating disulfide bonds into mesoporous silica frameworks to synthesize organic silica shells, the resulting structure enables disulfide bond cleavage under specific conditions (*e.g.*, in the presence of GSH), thereby enabling responsive drug release. In this study, disulfide bonds were hybridized into an inorganic silica layer to form an organic-inorganic mesoporous silica shell. This design allows disulfide bonds to undergo redox reactions in environments such as GSH solutions, triggering bond cleavage and subsequent degradation of the silica shell. This mechanism facilitates the selective and responsive release of drugs loaded within the mesopores.

**Fig. 4:**
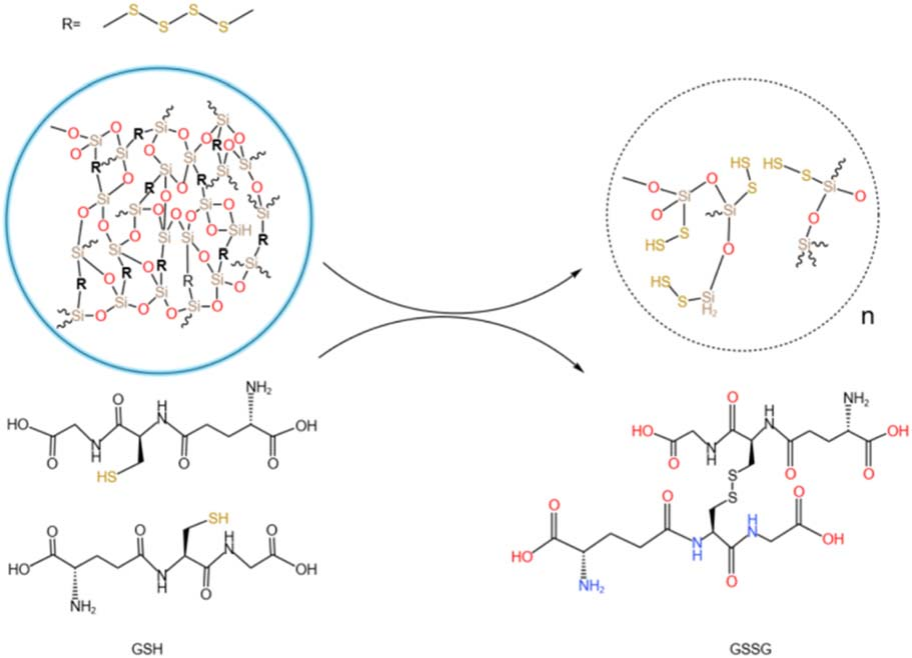
Degradation mechanism of GSH and disulfide bond oxidation–reduction reaction.

Tumors exhibit an acidic microenvironment with high GSH concentrations. GSH, a small molecular peptide containing thiol groups (–SH), functions as a reducing agent by participating in redox reactions with oxidized glutathione (GSSG), reducing it to GSH. Under acidic conditions, GSH demonstrates enhanced reducing capacity, as evidenced by its ability to reduce nitrite (NO_2_^−^) to nitric oxide (NO). This suggests that the reducing power of GSH is amplified in acidic conditions, accelerating disulfide bond cleavage. Fig. 4 illustrates the redox mechanism of disulfide bonds with GSH in acidic environments. GSH can undergo thiol-disulfide exchange reactions with disulfide bonds, generating new thiol groups and cleaving existing disulfide bonds. This reaction accelerates the degradation of the organic silica layer. This system is expected to enable specific redox-responsive drug release triggered by high GSH levels and acidic conditions.

**Fig. 5:**
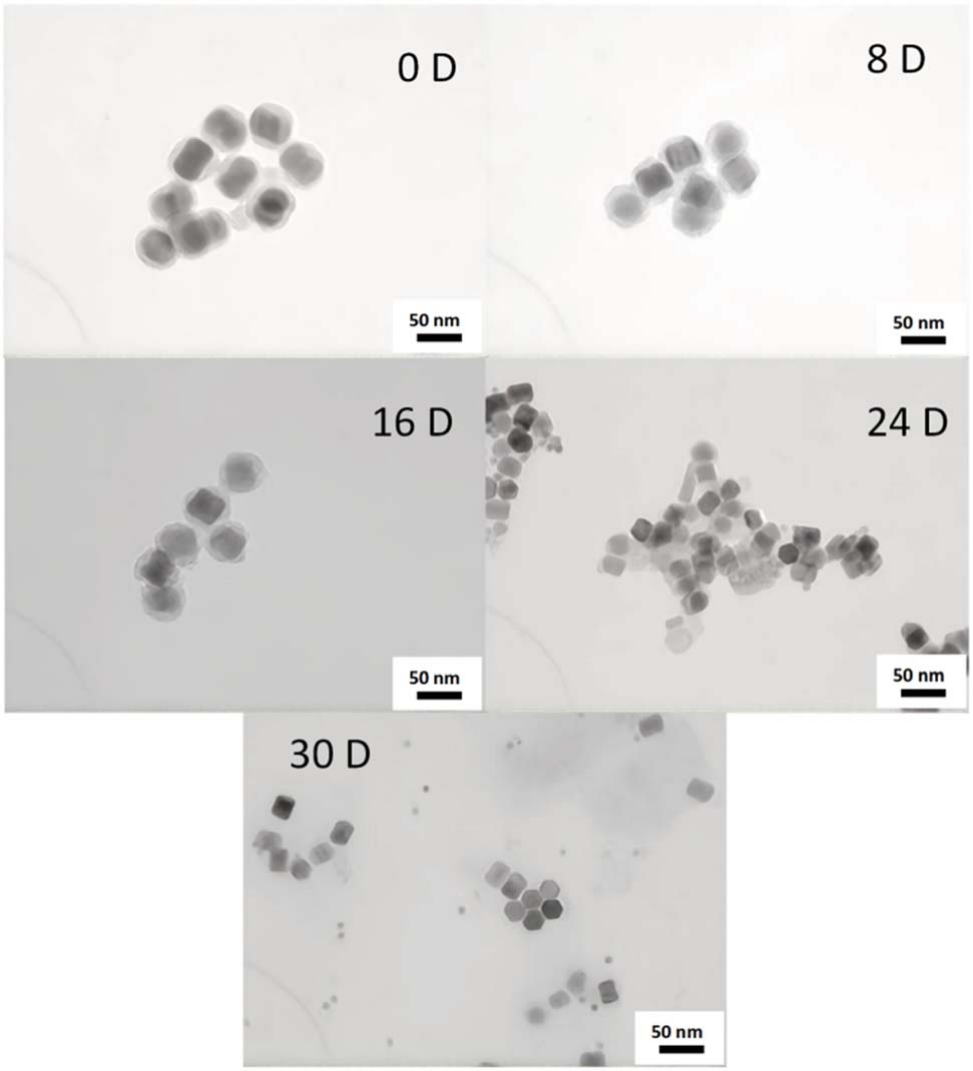
TEM images of 0, 8, 16, 24 and 30 days of degradation of UCNP@(s-s)mSiO_2_.

Fig. 5 presents TEM images of the nanocapsules at 0, 8, 16, 24, and 30 days, visually illustrating their degradation trends over time. Initially, the silica shell remained intact, and the particles exhibited uniform morphology. Upon exposure to an acidic GSH-rich environment, the silica shell of the nanocomposite particles initially showed localized swelling and minor degradation. As degradation progressed, the shell became thinner, and by the final stages, the UCNPs were fully exposed. Consistent with the proposed mechanism, the silica shell first interacts with GSH, initiating gradual degradation of the crosslinked network. The acidic environment (pH 5.0) not only enhances the reducing activity of GSH (as thiol groups are more prone to deprotonation into nucleophilic thiolate anions at low pH) but also destabilize the silica shell through protonation effects, accelerating structural disintegration. The degradation rate of the silica shell is significantly increased in acidic GSH conditions. Disulfide bonds (–s–s–) are cleaved by GSH reduction, disrupting the crosslinked structure of the shell. The degradation of the silicone layer indicates that the synthesized silicone has obvious degradation potential and is expected to respond to the tumor microenvironment in the future. The effective degradation of silicon layer not only optimizes the release efficiency of drug delivery systems, but also demonstrates significant advantages in safe metabolism *in vivo* due to its good biodegradability.

**Fig. 6:**
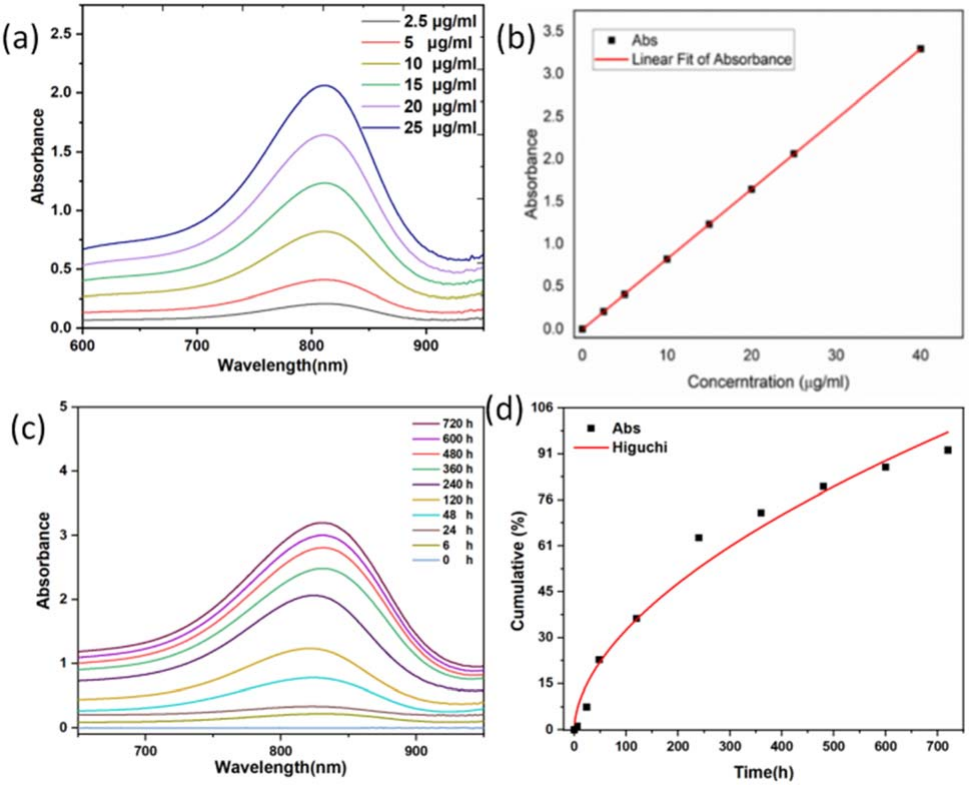
(a) UV-vis spectra of different silicic acid concentrations, (b) absorbance-silicon concentration relationship, (c) UCNPs@(s-s)mSiO_2_ ultraviolet spectra of molybdenum-silicon-blue at different degradation times, (d) Scatter plots of ultraviolet absorption intensity at 810 nm of molybdenum-silicon-blue at different degradation times, and the kinetic fitting curves of Higuchi model.

The degradation kinetics of the organic silica were investigated using UV-vis spectrophotometry based on the molybdenum silicate blue method.^48^ Fig. S5a illustrates the reaction mechanism. In acidic media, silicic acid (H_4_SiO_4_) reacts with ammonium molybdate ((NH_4_)_2_MoO_4_) to form the yellow silicon molybdate heteropolyacid (H_8_[Si(Mo_2_O_7_)_6_]). As the yellow product is unstable, it is reduced to blue silicomolybdenum blue (H_8_[Si(Mo_2_O_5_)(Mo_2_O_7_)_5_]) using ascorbic acid as a reducing agent. Fig. S5b shows photographs of silicic acid solution (left), silicon molybdate yellow solution (middle), and silicomolybdenum blue solution (right).Fig. 6a and b present the UV-visible absorption spectrum of the molybdenum-blue complex and its corresponding calibration curve at standard concentrations.

To evaluate the degradation process and predict degradation kinetics, various classical mathematical models were employed for kinetic fitting. The Higuchi equation, a release kinetics model, was used to describe drug release in controlled-release systems. This model provides a mathematical framework for quantitatively analyzing the diffusion and matrix dissolution processes of drug molecules in sustained-release systems, with a concise mathematical form and clear physical interpretation.^49,50^

The degradation kinetics were analyzed using the parameters listed in Table 1: *M*_*t*_ represents the cumulative silicic acid release at time *t*, and *M*_∞_ represents the cumulative silicic acid release at *t* = ∞. The characteristic kinetic constant is denoted as *k*. In the design and analysis of controlled-release systems, the release kinetics of active components was governed by multiple parameters, including the diffusion coefficient of drug molecules within the matrix, as well as adsorption and permeation processes. The Higuchi release kinetics model integrates these key factors into a mathematical framework, providing precise predictions and descriptions of drug release rates. The molybdenum silicate blue method used in this study serves as a means to evaluate the sustained release of silicic acid within the dialysis bag. As shown in Fig. 6c and d, the determined kinetic constant *k* was 0.01212, with a correlation coefficient *R*^2^ = 0.96158, indicating a high goodness-of-fit of the Higuchi model for the degradation behavior of the system.

**Table 1:** Higuchi model formula and release rate constant, intercept and correlation coefficient

Kinetic models	Kinetic equations	*K*	*R* ^2^
Higuchi	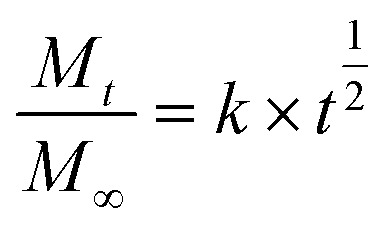	0.1212	0.96158

Compared to typical polymeric degradation systems, the degradation rate of the organic–inorganic hybrid silica-based material exhibited significant lag. Further analysis revealed that this difference arises from two critical factors. Firstly, the high glass transition temperature of the silica matrix enhances the material's structural stability. Secondly, the doping ratio of organic components, such as disulfide bonds, has not reached an optimal threshold, resulting in a relatively high activation energy barrier for chemical degradation.^51,52^ Notably, the release kinetics data showed a high linear correlation with Fick's law of diffusion (slope deviation <5%), confirming that drug release in this system is primarily governed by a diffusion mechanism driven by concentration gradients.

### 3.4 Loading DOX into UCNPs@(s-s)mSiO_2_@Azo

**Fig. 7:**
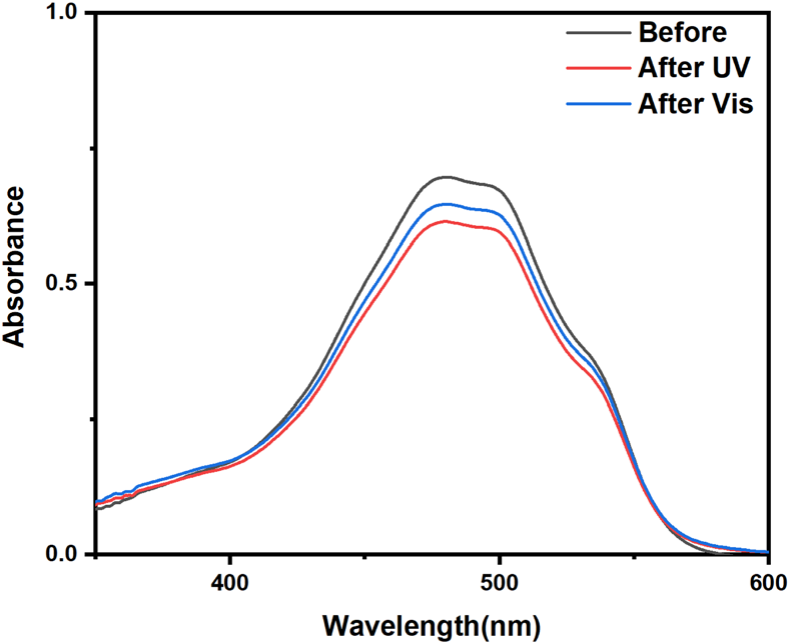
UV visible absorption spectra of the supernatant after drug loading by different methods.

DOX was used as a model drug to evaluate the drug-loading and release behavior of UCNPs@(s-s)mSiO_2_@Azo nanocomposite particles. Fig. 7 shows the UV-vis spectra of the supernatant from centrifuged solutions before and after drug loading under different conditions. The results indicate a significant decrease in absorbance at 480 nm after drug loading, confirming successful DOX loading onto UCNPs@(s-s)mSiO_2_@Azo composite nanoparticles.

Azo groups undergo *trans* to *cis* isomerization upon UV irradiation. The *cis* isomer has higher polarity and a bent molecular structure, which expands the mesoporous silica channels and reduces steric hindrance for drug molecules, thereby enhancing drug loading efficiency. In contrast, under visible light, the pores contain *trans*–*azo* groups, which increase steric hindrance and might reduce drug loading by preventing drug entry, though they also minimize drug leakage during loading.

A standard DOX concentration–absorbance curve at 480 nm (Fig. S6) was used to calculate drug-loading efficiencies. The loading efficiency of UCNPs@(s-s)mSiO_2_@Azo nanocapsules was 12.2 wt% under UV light and 8.1 wt.% under visible light. The UV-irradiated system showed higher loading efficiency than other UCNPs-based photoresponsive systems. This study employed ultraviolet light irradiation as the condition for drug loading. For instance, Wang *et al.*^53^ reported a 10 wt% DOX loading efficiency for UCNPs@SP-MA/MAA nanoparticles.

### 3.5 Controlled release behavior of DOX- UCNPs@(s-s)mSiO_2_@Azo Nanocapsules

**Fig. 8:**
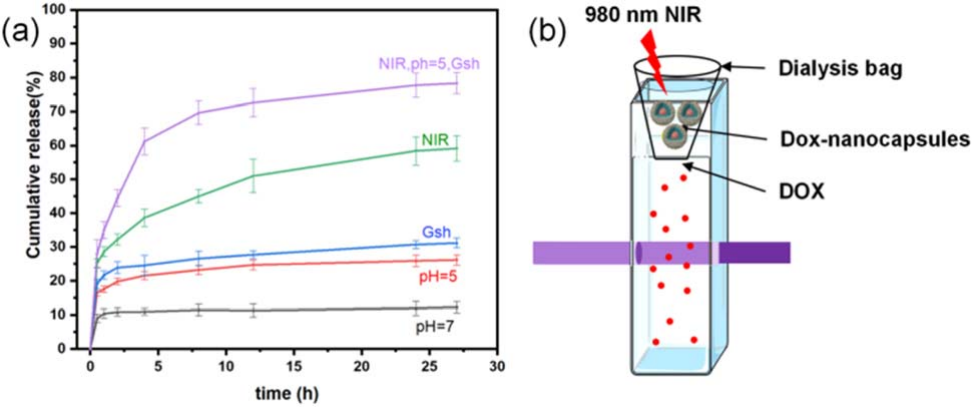
(a) DOX cumulative release of DOX-UCNPs@(s-s)mSiO_2_@Azo nanocapsules and (b) schematic diagram of the setup used to measure the controlled release of nanocapsules.

As shown in Fig. 8a, in pH 7.4 buffer, the cumulative DOX release from UCNPs@(s-s)mSiO_2_@Azo nanoparticles was only 12.2% (±1.61%) after 27 hours, indicating stable drug encapsulation under neutral conditions due to the grafted azobenzene groups hindering drug diffusion. In pH 5.0 buffer, the cumulative release increased to 26.1% (±1.51%) after 27 hours. Protonation of azobenzene derivatives in acidic conditions alters their conjugated system, weakening interactions with mesoporous silica and promoting drug release. In neutral buffer with high GSH (10 mM), the cumulative release was 31.2% (±1.46%) after 27 hours. As shown in Fig. 4, GSH reduces disulfide bonds (–s–s–) in the mesoporous silica to thiols (–SH), disrupting crosslinks and opening pores for DOX release. Under neutral conditions with near-infrared (NIR) irradiation, the cumulative release reached 59.1% (±3.72%) after 27 hours. UCNPs convert NIR to UV/visible light, triggering photoisomerization of the azobenzene derivative AB-TPI. The *cis*-isomer's smaller size or polarity change opens pores, and the continuous isomerization acts as a “molecular stirrer,” accelerating DOX release with spatiotemporal precision.

In weakly acidic conditions (pH 5.0) with high GSH (10 mM) and NIR irradiation, the cumulative release was 78.3% (±3.11%) after 27 hours. This simulated tumor microenvironment accelerates disulfide bond cleavage *via* redox reactions and enhances degradation of the silica shell. Simultaneously, UCNPs convert NIR to UV/visible light, driving AB-TPI isomerization. The combined photoresponsive and redox effects further promote drug release.

To analyze drug release from different matrices, the Baker-Lonsdale model for spherical matrices was applied to quantify DOX release from nanocapsules under various conditions,^54–56^ as described below:
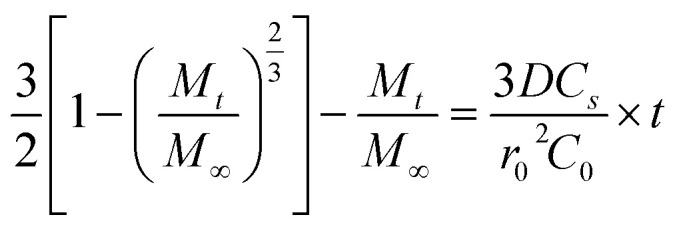
where *M*_*t*_ and *M*_∞_ are the amounts of DOX released at time *t* and *t* = ∞, respectively. *M*_*t*_/*M*_∞_ is the cumulative release of DOX, *D* is the diffusion coefficient of DOX in the nano drug delivery system, *r*_0_ is the radius of the nanocomposite particles, *C*_*s*_ is the solubility of DOX, and *C*_0_ is the initial concentration of DOX.

**Fig. 9:**
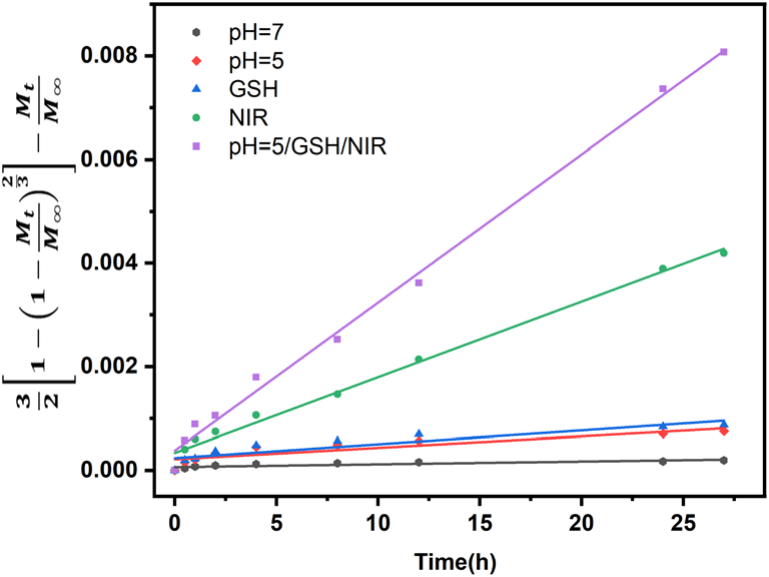
Linear regression analysis using the Baker-Lonsdale model of DOX from nanocapsules as a function of time.

Fig. 9 shows DOX release from the Baker-Lonsdale model under different pH levels and NIR exposure times, with release rate constants (3*DC*_*s*_/*r*_0_2*C*_0_) and *R*^2^ correlation coefficients as follows: 4.04 × 10^−6^ and 0.72 (pH = 7.4); 6.31 × 10^−6^ and 0.89 (pH = 5); 7.14 × 10^−6^ and 0.90 (with GSH); 1.41 × 10^−4^ and 0.99 (with NIR); and 2.81 × 10^−4^ and 0.99 (pH = 5, GSH, NIR). Under the nanocapsules system, *C*_0_=*C*_*s*_. The diffusion coefficient *D* under NIR is two orders of magnitude larger than without NIR, indicating faster drug release. With pH = 5.0, GSH, and NIR, *D* doubles again. This shows that under simulated tumor conditions (pH = 5.0, GSH) and NIR irradiation, the nano-theranostic agent releases drugs faster through photo-redox dual-responsive mechanisms.

### 3.6 Assessing *in vitro* drug release behavior

**Fig. 10:**
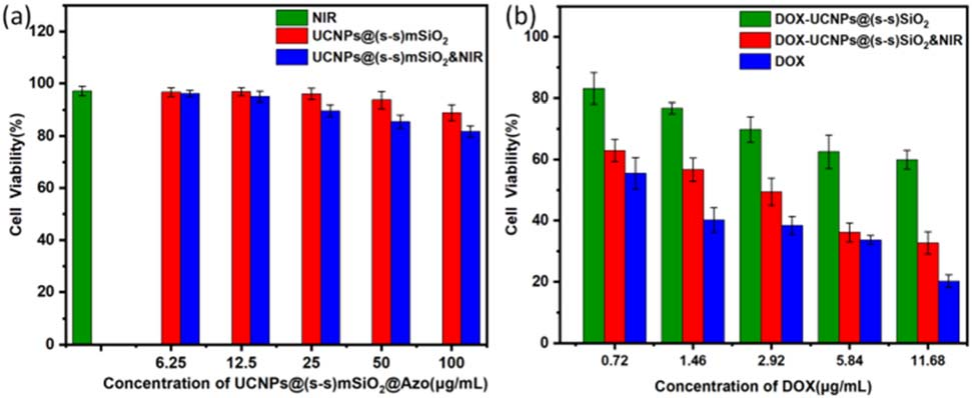
(a) Nanocapsules under different conditions o cell survival rate of nanocapsules; (b) nanocapsules under different conditions cell survival rate of nanocapsules.

Since the cytotoxicity of nanomedicine carriers is an important prerequisite and key factor determining their further biological applications, 4T1 cells were stimulated by NIR irradiation using the MTT method, and the cytotoxicity of nanocapsules was measured *in vitro*. As shown in Fig. 10a, near-infrared irradiation and nanocapsules had little effect on cell viability. The results show that DOX-UCNP@(s-s)mSiO_2_@Azo nanocomposite particles have good biocompatibility under near-infrared radiation, meeting the biological application requirements as a potential drug carrier. Fig. 10b shows 4T1 cell viability, DOX and nanocapsules exposed to near-infrared radiation at different intensities in the medium. When the nanocapsules were raised to 25 µg mL^−1^, no significant cell death was observed after 24 h of incubation with the drug-loaded complexes, even without near-infrared stimulation. When the concentration of the drug-loaded composite nanoparticles was further increased to 100 µg mL^−1^ and combined with near-infrared irradiation, the cell survival rate sharply decreased to 32.64% (±3.63%), showing a significant difference compared with the non-illuminated group at the same concentration. It is worth noting that in the drug-free controlled experiment, as shown in Fig. 10a, the synergistic effect of near-infrared illumination and the blank vector did not cause significant cytotoxicity, which strongly confirmed that the significant change in cell mortality was due to the drug release mechanism of near-infrared and endogenous reactions rather than the direct effect of the vector itself or physical stimulation.

**Fig. 11:**
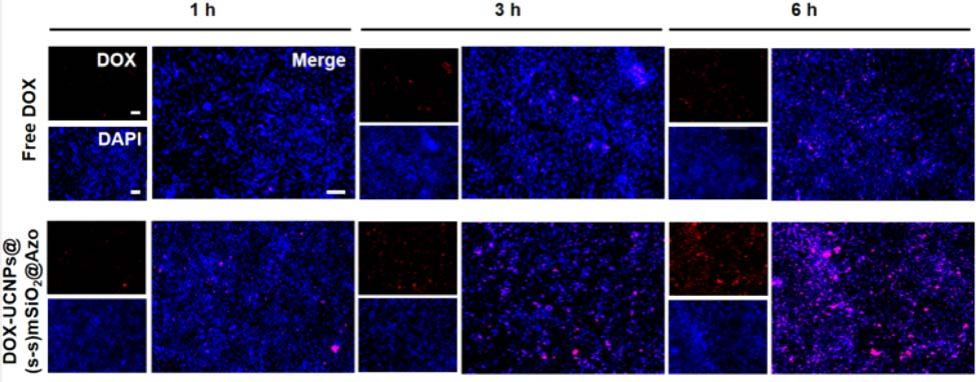
Fluorescence images of free DOX nanoparticles and nanocapsules incubated with 4T1 cells for different durations. The images show DOX fluorescence (red), cell nuclei stained with Hoechst 33 342 (blue), and a merged image of DOX fluorescence and nuclear staining (scale bar: 100 µm).

Due to the fluorescence of DOX, the internalization of nanocapsules could be easily visualized by fluorescence microscopy for the qualitative uptake of 4T1 cells. As shown in Fig. 11, confocal laser microscopy images of nanocapsules exposed to 4T1 cells for 1, 3, and 6 hours revealed that the intracellular accumulation of nanocapsules increased over time. It indicates that 4T1 cells successfully uptake nanocomposite particles. Nanocapsules particles internalize a higher amount of DOX and are superior to free DOX.

## 4. Conclusions

In this paper, we prepared nanocapsules with multiple drug-controlled release responses. The drug loading rate under UV irradiation in pH 7.4 PBS was 12.2 wt%. In an acidic, high-GSH environment, the organic silica shell degrades almost completely, and the Higuchi model was used to construct the degradation kinetics of the organic silica shell. Under near-infrared light, the smart photoresponsive agent showed good controlled drug release, and the specific redox degradation of the particles improved drug release efficiency. This study focuses on the photoresponsive, multi-stimuli responsive drug release of nanocapsules under various conditions and calculates the diffusion coefficient under different conditions using the Baker-Lonsdale model. These nanocomposites offer a promising method for targeted tumor therapy and provide a new platform for studying stimuli-responsive drug delivery systems.

## Author contributions

Xiaotao Wang: conceptualization, methodology, data curation, writing – original draft. Zhihao Bi: methodology. Yonggui Liao: investigation, formal analysis, conceptualization. Xuefeng Li: revise.

## Conflicts of interest

There are no conflicts to declare.

## Supplementary Material

RA-016-D5RA07668D-s001

## Data Availability

The data supporting this article have been included as part of the supplementary information (SI). Supplementary information: Fig. S1–S3, SEM images of nanocapsules. Fig. S4, UV-vis absorption and infrared spectra of nanocapsules. Fig. S5, pictures of different reaction solutions. Fig. S6, UV-vis spectra of drug loading and release. Fig. S7, UV-vis spectra of drug release under different conditions. See DOI: https://doi.org/10.1039/d5ra07668d.
