# Supplementary material for: Multi-responsive upconversion/organic porous silicon nanocomposite for controlled drug release via NIR irradiation and tumor microenvironment stimuli
Source: RSC Adv. 2026 Jan 23;16(6):5168–78. doi: 10.1039/d5ra07668d (PMC12829441; doi:10.1039/d5ra07668d)
Supplement: RA-016-D5RA07668D-s001 [file RA-016-D5RA07668D-s001.pdf]

Supplementary Information (SI) for RSC Advances.
This journal is © The Royal Society of Chemistry 2025

Electronic Supplementary Information (ESI)

Multi-Responsive Upconversion/Organic Porous Silicon Nanocomposite for Controlled Drug Release via NIR Irradiation and Tumor Microenvironment Stimuli

XiaotaoWang[1], Zhihao Bi[1], Yonggui Liao[2]* Chak-Yin Tang[3], Xuefeng Li [1]*, Wing-Cheung Law[3]*

[1]Hubei Provincial Key Laboratory of Green Materials for Light Industry, Collaborative Innovation Center for Green Light-weight Materials and Processing, School of Materials Science and Engineering, Hubei University of Technology, Wuhan, 430068, China

[2]Key Laboratory for Material Chemistry for Energy Conversion and Storage, Ministry of Education, Hubei Engineering Research Center for Biomaterials and Medical Protective Materials, Hubei Key Laboratory of Material Chemistry and Service Failure, School of Chemistry and Chemical Engineering, Huazhong University of Science and Technology, Wuhan 430074, China

[3]Department of Industrial and Systems Engineering, The Hong Kong Polytechnic University, Hung Hom, Kowloon, Hong Kong, China

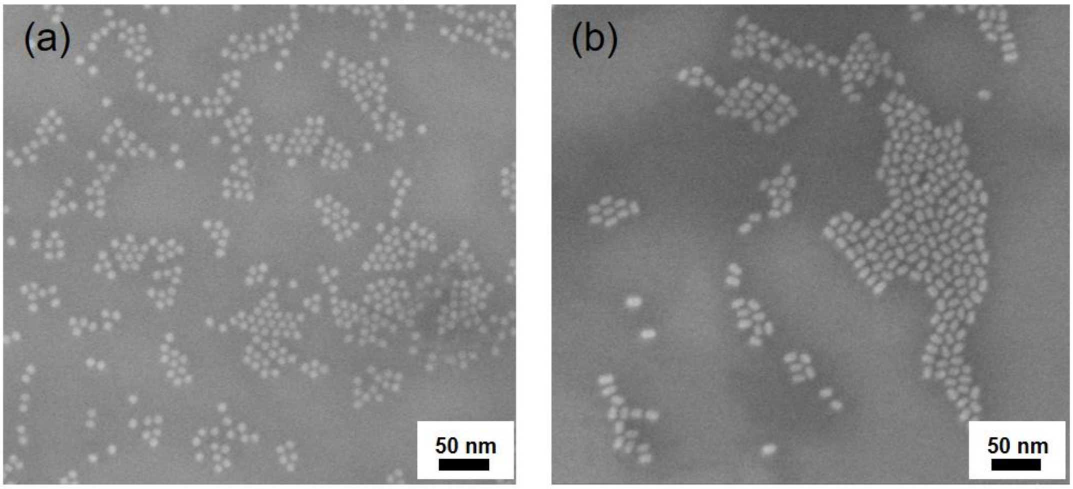

Figure S1.(a)SEM images of $NaYF_4$: Yb^{3+}/Tm^{3+};(b)SEM images of $NaYF_4$: Yb^{3+}/Tm^{3+}@$NaYF_4$: Nb^{3+}/Ym^{3+}.

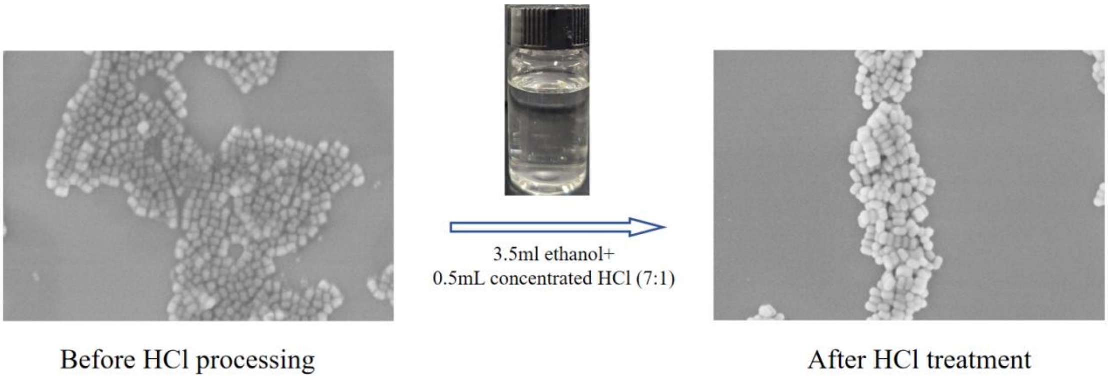

Figure S2. Comparison of the Operation and SEM Image of Removing Oleic Acid Ligand with Hydrochloric Acid Ligand.

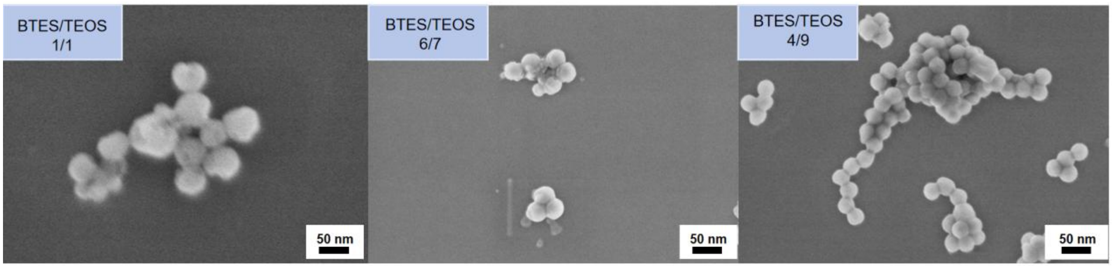

Figure S3. SEM images of UCNPs@(s-s)mSiO_2 nanoparticles with different silicon source ratios

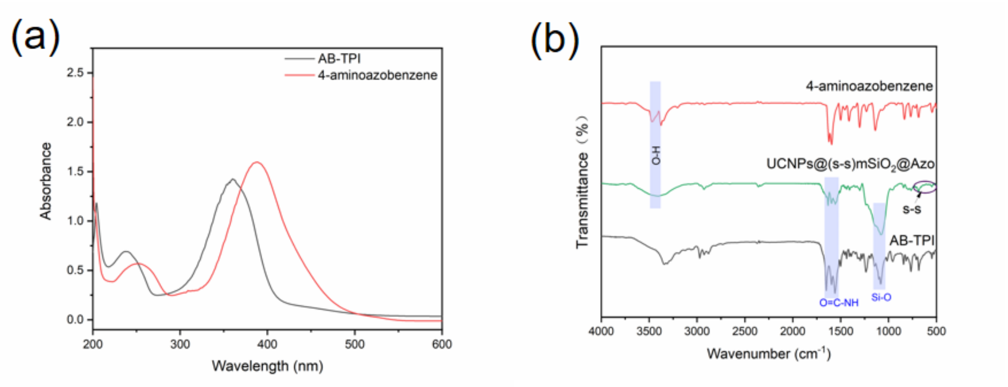

Figure S4. (a) UV-Vis absorption peaks of 4-aminoazobenzene and AB-TPI. (b) Infrared spectra of 4-aminoazobenzene, AB-TPI, UNCPs@UCNPs, and UCNP@(s-s)mSiO_2.

As shown in Figure S4a, the absorption peak of the synthesized AB-TPI shifted from 400 nm to 350 nm under UV light, confirming its ability to absorb ultraviolet radiation.FTIR results (Figure S4b)revealed characteristic bands: a peak at 1548 cm^{-1}attributed to the stretching vibration of-NH-CO-NH-formed after the coupling reaction between the silane coupling agent and the amino group, a band at1502 cm^{-1}corresponding to-NH-stretching vibrations, and a peak at3300 cm^{-1}assigned to the diazo group.

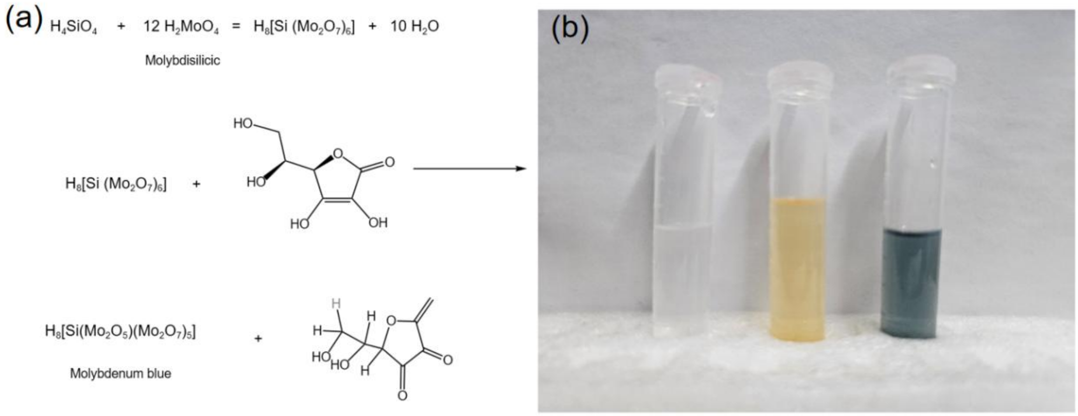

Figure S5. (a) Reaction mechanism of molybdenum silicon blue. (b) Pictures of silicic acid solution, molybdenum silicate yellow, and molybdenum silicate blue from left to right.

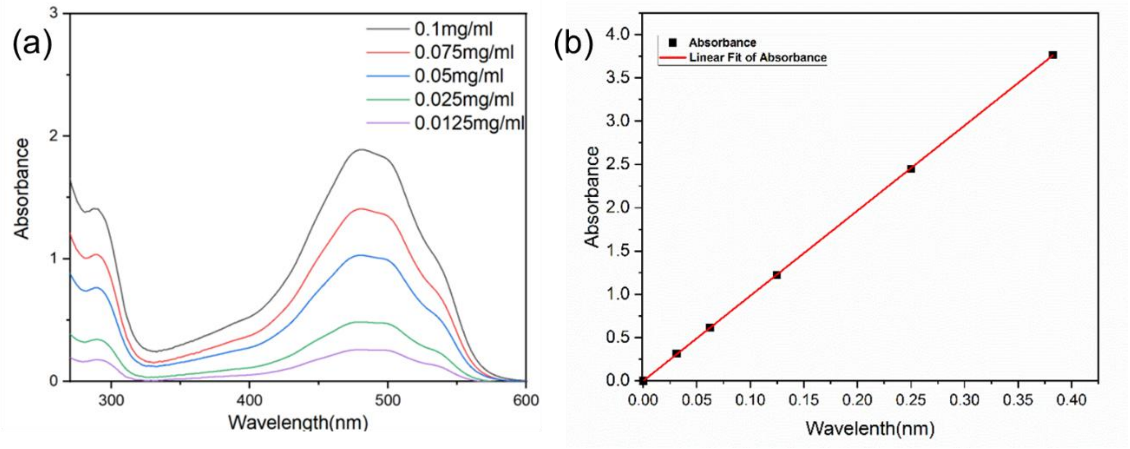

Figure S6. (a) UV spectra of DOX before and after drug loading. (b) Absorbance - concentration relationship of DOX.

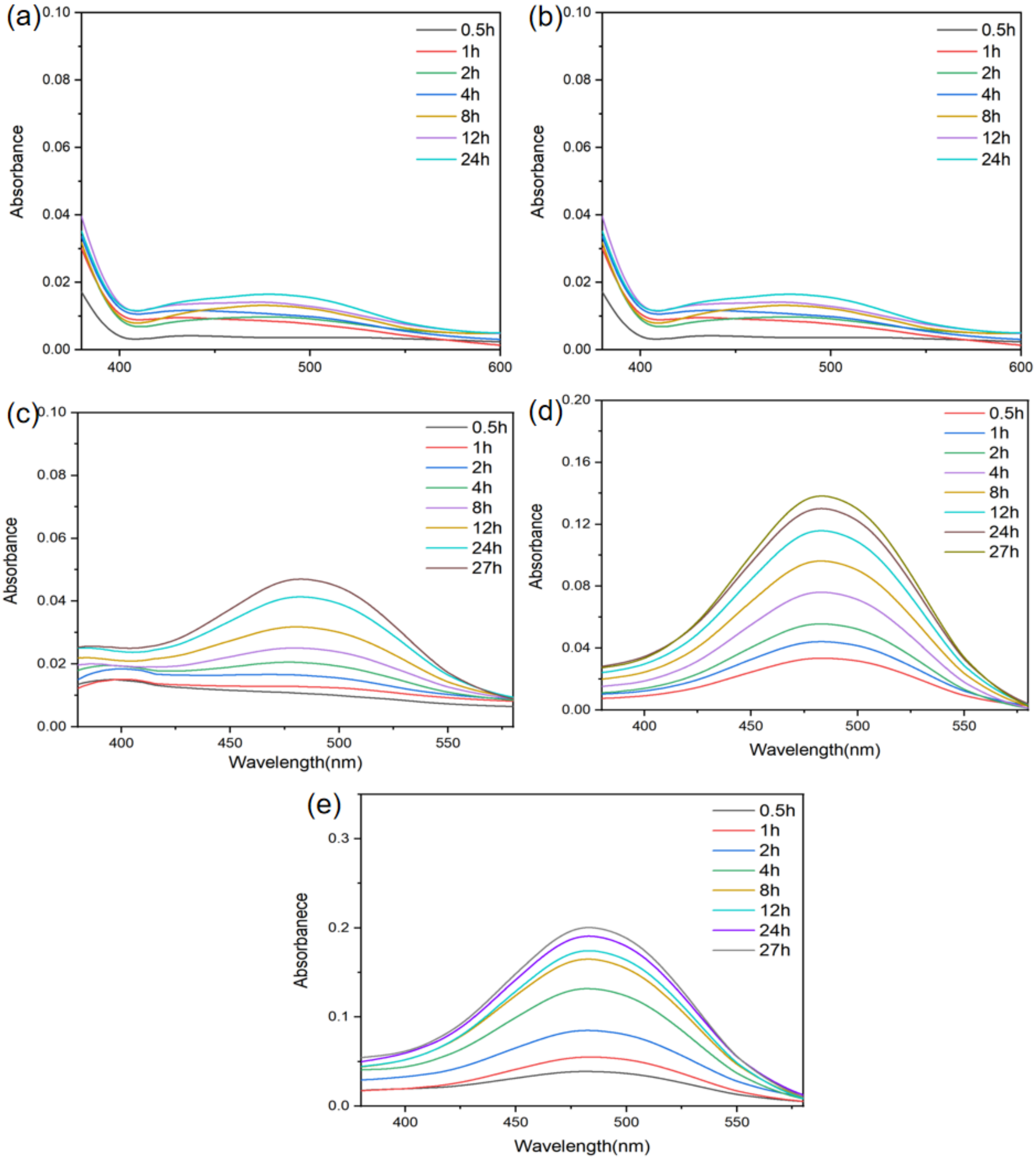

Figure S7. Controlled release under different conditions: (a) pH=7, (b) pH=5, (c) GSH, (d) NIR and (e) pH=5/GSH/NIR.
